# Molecular Mechanisms of Intestinal Protection by *Levilactobacillus brevis* 23017 against *Salmonella typhimurium* C7731-Induced Damage: Role of Nrf2

**DOI:** 10.3390/microorganisms12061135

**Published:** 2024-06-01

**Authors:** Ziqi Shi, Yongchao Nan, Xinyao Zhou, Wenzhi Zhang, Zheng Zhang, Chuankun Zhang, Haoyuan Duan, Junwei Ge, Lili Zhao

**Affiliations:** 1 State Key Laboratory for Diagnosis and Treatment of Severe Zoonotic Infectious Diseases, Key Laboratory for Zoonosis Research of the Ministry of Education, Institute of Zoonosis, and College of Veterinary Medicine, Jilin University, Changchun 130062, China; szq13115449695@163.com; 2 Heilongjiang Provincial Key Laboratory of Zoonosis, College of Veterinary Medicine, Northeast Agricultural University, Harbin 150030, China; nanyongchao365@163.com (Y.N.); s210601031@neau.edu.cn (X.Z.); b20060122@neau.edu.cn (W.Z.); zhangzheng201704@163.com (Z.Z.); b230601006@neau.edu.cn (C.Z.); elix349@163.com (H.D.)

**Keywords:** *Levilactobacillus brevis* 23017, *Salmonella typhimurium* C7731, oxidative stress, SIgA, Nrf2 signaling pathway

## Abstract

The treatment and prevention of pathogenic diseases by lactic acid bacteria (LAB) has attracted more and more attention. As a special LAB, *Levilactobacillus brevis* (*L. brevis*) has relatively less research on its antibacterial infection in vivo, and its protective effect and mechanism still need to be fully studied. In this study, we selected *L. brevis* 23017, which can regulate the intestinal immunity of the host animal and resist pathogen infection, to evaluate its protective role and potential molecular mechanisms in the mouse model of *S. typhimurium* C7731 infection. As expected, we confirmed that *L. brevis* 23017 reduced the diarrhea rate and increased the daily weight gain and survival rate of the mouse model, and inhibited *S. typhimurium* colonization in the jejunum and liver. It also reduced the level of oxidative damage and protected the integrity of intestinal tissue by increasing the activity of intestinal antioxidant enzymes (SOD, GSH-Px and T-AOC). From the perspective of intestinal mucosal barrier injury and repair, it was confirmed that *L. brevis* 23017 could increase the expression levels of intestinal tight junction proteins (ZO-1 and OCLN). Our research results also show that *L. brevis* 23017 inhibits the inflammatory response and promotes the occurrence of cellular immunity in the body by promoting the increase in IL-10 and inhibiting IL-13 in serum and intestinal tissue. Notably, *L. brevis* 23017 increased total secretory immunoglobulin A (SIgA) levels in the intestine, which were closely associated with elevated levels of IL-5, IL-13, pIgR, j-chain, and IgAα-chain. In addition, *L. brevis* 23017 increased the expression of antioxidant proteins Nrf2, NQO1, and HO-1 associated with Nrf2 signaling to inhibit intestinal oxidative damage. This mechanism may be responsible for its protective effect against *S. typhimurium*-infected intestine. Our study provides new evidence and theoretical support for the analysis of the anti-bacterial infection effect and mechanism of *L. brevis*, which will contribute to the development of *L. brevis* and the treatment of pathogenic bacteria intestinal infection.

## 1. Introduction

Lactic acid bacteria (LAB) are one of the most commonly used probiotics [1]. LAB exhibit excellent characteristics that include acid and bile resistance, adhesion, and bacteriostatic properties [2,3]. Attributes of LAB include stabilizing intestinal flora, improving lactose intolerance, increasing nutrient utilization, improving digestion, and stimulating the immune system [4,5,6,7]. Many LAB products have been developed for human and animal husbandry applications, and are mainly used to treat and prevent disease [8]. Studies of LAB have especially included gastrointestinal infections [9] and the potential of LAB to curb bacterial infections [10].

The prowess of LAB against bacterial infections could involve promotion of the secretion of mucin by intestinal epithelial cells, which inhibits adhesion of pathogenic bacteria to intestinal epithelial cells and the consequent colonization of the intestinal lining [11]. Other mechanisms could contribute to the reinforcement of the intestinal barrier function [12,13]. In vitro cellular experiments have demonstrated that LAB can increase the transmembrane resistance of monolayer cells, inducing the secretion of occludin (OCLN), claudin, zonula occludens-1 (ZO-1), and ligand adhesion factors to reduce intercellular permeability and protect the intestinal mucosal barrier [14]. Furthermore, LAB can effectively inhibit the production of pro-inflammatory cytokines, positively regulate the secretion of anti-inflammatory cytokines, and modulate the intestinal immune response [15,16,17]. Evidence has also indicated variability in the properties and characteristics of different LAB; thus, the effect of specific LAB on specific diseases or pathogens requires more research to evaluate their probiotic potential [18,19].

*Levilactobacillus brevis* has been studied less than other LAB as a probiotic strain. Although many studies have addressed the antibacterial effects in vitro, data of the in vivo antimicrobial activity of *L. brevis* are limited, as are in vitro evidence for the antimicrobial effect of *L. brevis* against *Salmonella*. We have previously described the protective effects of a select probiotic strain, *L. brevis* 23017, which showed protective effects against *Yersinia enterocolitica* infection in a mouse model, involving the regulation of the mitogen-activated protein kinase and nuclear factor-kappa B pathways [20]. Important roles were ascribed to antioxidant enzyme production and the stimulated production of secretory immunoglobulin A (SIgA). However, the nature and mechanism of the antibacterial effects of *L. brevis* in vivo are unclear.

Nuclear factor erythroid 2-related factor 2 (Nrf2) is a constituent of the well-characterized ubiquitin-dependent signaling pathway that responds primarily to oxidative stress and is thought to be a major regulator of protection against cellular stress [21]. Its major antioxidant genes include NADPH quinine oxidoreductase 1 (NQO1) and heme oxygenase-1 (HO-1) [22]. NQO1 reduces the detoxification and associated toxicity of free radicals and exogenous substances [23]. HO-1 is responsible for the catalytic breakdown of heme to biliverdin, which is further converted to bilirubin [24]. The specific antioxidant mechanisms of LAB as antioxidant microbial agents need to be analyzed by in vivo and in vitro assays [25]. A recent investigation of LAB activity against oxidative damage caused by bacterial infection has attracted much attention [26]. Other authors have demonstrated the protection by lactobacilli against epithelial cell injury through the Nrf2 pathway, implicating this pathway as a unique system for non-immune perception. Dou et al. [27] demonstrated in vitro that *Clostridium butyricum* protects IPEC-J2 cells from enterotoxigenic *Escherichia coli* (ETEC) K88-induced oxidative damage by activating the Nrf2/ARE signaling pathway. Wang et al. [28] demonstrated that *Bacillus amyloliquefaciens* SC06 protects against hydrogen peroxide (H_2_O_2_)-induced oxidative damage to IPEC-1 cells via the Nrf2/Keap1 signaling pathway. However, the mechanism of the in vivo antioxidant activity of LAB is still unclear. The protective effects and mechanisms of LAB against oxidative stress induced by bacterial infections require clarification.

In this study, we evaluated the protective effects of *L. brevis* 23017 against *S. typhimurium* C7731 infection in a mouse model by observing body weight changes, disease activity index (DAI) score, bacterial load, antioxidative capability, inflammation, and intestinal damage. The protective mechanisms were revealed by investigating immune-related functional genes and cytokines, particularly the polymeric Ig receptor A (pIgR), J-chain, and IgA α-chain structural factors associated with SIgA. Finally, the Western blot assay was used to assess the expression levels of antioxidant proteins associated with the Nrf2 signaling pathway. Our results show that activation of the Nrf2 signaling pathway plays an important role in the protective effect of *L. brevis* 23017 against *S. typhimurium* C7731 infection.

## 2. Materials and Methods

### 2.1. Ethics Statement

The Ethical Committee of Northeast Agricultural University (NEAUE), Harbin, China, the Institute approved all scientific experiments. All applicable international and national guidelines for the care and use of animals in experiments were followed and approved (NEAUEC20210326, 30 March 2021).

### 2.2. Bacterial Strains

*L. brevis* 23017 was isolated, identified, and preserved by Heilongjiang Provincial Key Laboratory of Zoonosis. Strain 23017 was confirmed to have good antibacterial and efficient antioxidant and anti-inflammatory abilities [20]. It was cultured in MRS medium (Hope Bio-Technology Co., Ltd., Qingdao, China) for 14 h at 37 °C. The resulting culture that typically contained 1 × 10^9^ colony forming units (CFU)/mL [29] was centrifugated at 4000× *g*. The supernatant was discarded and the pellet of bacteria resuspended in 200 µL of phosphate-buffered saline (PBS).

*S. typhimurium* C7731 was provided by Professor Na Dong, College of Animal Science and Technology, NEAU. Cells were cultured in Luria Bertani medium (Hope Bio-Technology) for 12 h at 37 °C. Preliminary experiments used 1 × 10^8^ CFU/mL *S. typhimurium* C7731 in the oxidative damage model as the minimum pathogenic bacterial infection. Following centrifugation at 4000× *g,* the supernatant was discarded and the bacterial pellet resuspended in 200 µL PBS.

### 2.3. Mice

Twenty BALB/c female mice (20–22 g, 6–8 weeks old) were purchased from the Experimental Animal Center of the Second Hospital of Harbin Medical University. The mice were housed in a controlled environment (22 °C, alternating 12 h periods of light and dark, with free access to food and water).

### 2.4. Experimental Design

**Figure 1:**
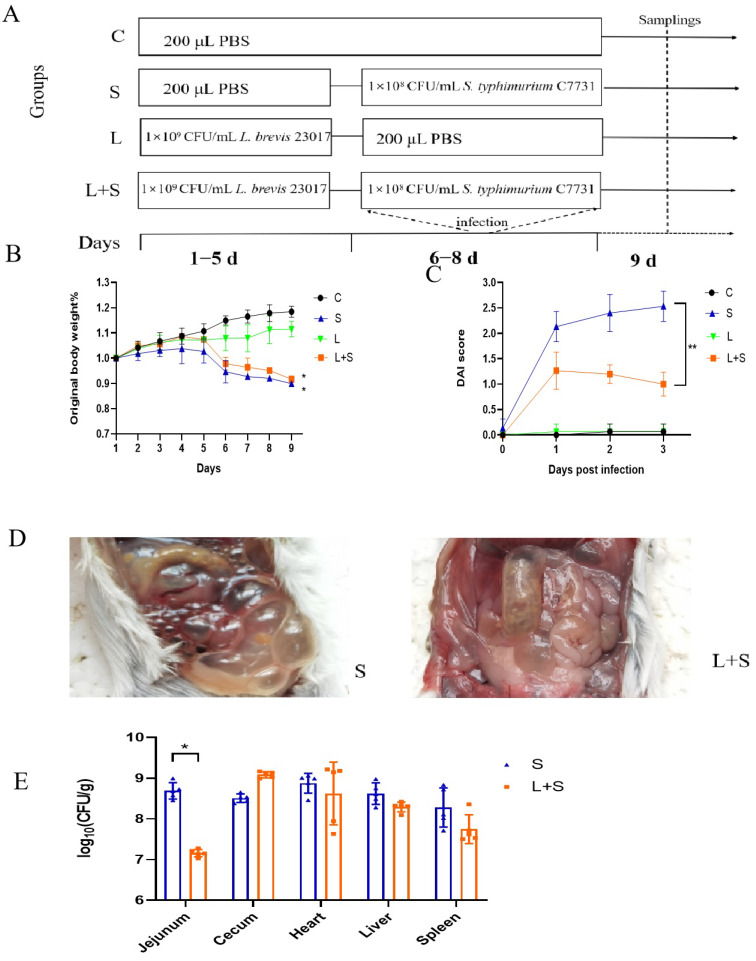
Study design and protective effect of *L. brevis* 23017 against *S. typhimurium* C7731 intestine infections. (**A**) Experimental protocol in mice. (**B**) Changes in body weight. (**C**) DAI scores after *S. typhimurium* C7731 infection. (**D**) Bacterial load in tissues and organs. (**E**) Gross pathological changes in the intestinal tract of the S and L+S groups. Means were considered to be significantly different at *p* < 0.05 (*) and highly significantly different at *p* < 0.01 (**).

The mice were distributed randomly into four groups. In the control group, gavage with 200 μL of PBS was administered during the experimental period for 1–8 d. These mice were fed normally and observed. In the S group, 200 μL PBS was administered on days 1–5, followed by gavage with 1 × 10^8^ CFU *S. typhimurium* C7731 on days 6–8 (this gavage was also used for the remaining groups). In the L group, 1 × 10^9^ CFU *L. brevis* 23017 was administered on days 1–5, followed by the gavage. Finally, in the L+S group, 1 × 10^9^ CFU *L. brevis* 23017 was administered on days 1–5, followed by the gavage. On day 9, all mice were fasted for 12 h and then executed. Samples were collected from each mouse for subsequent experiments. The specific experimental grouping design is shown in Figure 1A, and the nutrient and energy composition of the standard mouse chow is shown in Supplementary Table S1. Every mouse was monitored daily for weight loss, stool characteristics, and bleeding during the experiment. The DAI was assessed for each mouse as previously described [30,31].

### 2.5. Determination of S. typhimurium C7731 CFUs

Culture and identification of *S. typhimurium* C7731 were performed as previously described [32]. In brief, 0.1 g of mouse jejunum, cecum, heart, liver, and spleen were suspended in sterile PBS (1:10 *w*/*v*) and homogenized. Aliquots (0.1 mL) of each homogenate were plated on MacConkey agar medium and incubated at 37 °C. The total number of *S. typhimurium* C3371 colonies on the plates was determined.

### 2.6. Detection of Cytokines by Enzyme-Linked Immunosorbent Assay (ELISA)

The levels of the interleukin IL-10, IL-5, and IL-13 cytokines in sera were measured by ELISA following the kit instructions (Boster Biological Technology Co., Ltd., Wuhan, China).

### 2.7. Antibody Detection

Total SIgA in intestinal mucus samples was measured by ELISA following the kit instructions (Sangon Biotech (Shanghai) Co., Ltd., Shanghai, China).

### 2.8. Histopathological Examinations

The duodenum, ileum, and liver were fixed in 10% paraformaldehyde at room temperature for at least 48 h. Analyses of pathological sections were performed by Wuhan Servicebio Technology Co., Ltd. (Wuhan, China).

### 2.9. Antioxidant Activity Assay

Duodenal and jejunal samples were collected from each mouse. Malondialdehyde (MDA), superoxide dismutase (SOD), glutathione peroxidase (GSH-Px), and total antioxidant capacity (T-AOC) were measured in each tissue homogenate using the correspondent test kits (Nanjing Jiancheng Bioengineering Institute, Nanjing, China).

### 2.10. Real-Time Quantitative PCR (RT-qPCR)

RT-qPCR analysis was performed as previously described [33]. Due to the purpose of this study, some adjustments were made, with the duodenum and jejunum used as the intestinal tissues. RNA was extracted from the tissues with TRIzol reagent. (TaKaRa Biotechnology, Dalian, China) based on the manufacturer’s instructions. Total cellular RNA was used as a template and β-actin was used as an internal reference gene. Nrf2, NQO1, HO-1, pIgR, J-chain, IgA α-chain, ZO-1, OCLN, IL-10, IL-13, cyclooxygenase 2 (COX-2), and induced nitric oxide synthase (iNOS) mRNA expression levels were measured using the G 7500 Real-Time PCR System (Applied Biosystems, Franklin Lakes, NJ, USA). The Livak method (2^−ΔΔCT^ method) was used to calculate the fold change compared to β-actin gene controls. The RT-qPCR primers are shown in Supplementary Table S2.

### 2.11. Western Blot Analysis of Nrf2

The Western Blot assay was performed as previously described [33]. Briefly, tissue sections prepared from jejunum samples collected from each mouse were exposed to the following rabbit primary antibodies to glyceraldehyde 3-phosphate dehydrogeanse4 (GADPH), Nrf2, NQO1, HO-1, and Kelch-like ECH-associated protein 1 (Keap1) (all 1:2000 dilutions and all from Sigma-Aldrich, St. Louis, MO, USA). Horseradish peroxidase-conjugated secondary antibodies (1:5000, ABclonal, Woburn, MA, USA) were added and the blots were incubated at 25 °C. The sections were and observed by enhanced chemiluminescence (Beyotime Biotechnology, Shanghai, China).

### 2.12. Statistical Analysis

Statistical analysis was performed using GraphPad Prism 8.0.1 software (GraphPad, San Diego, CA, USA). All results are expressed as mean ± standard deviation. The data from the different groups were analyzed by one or two-way ANOVA. *p* < 0.05 and < 0.01 was significant and highly significant, respectively.

## 3. Results

### 3.1. Improvements in DAI Score and Clinical Symptoms by L. brevis 23017

Body weight changes of mice in the four groups are shown in Figure 1B. The body weight of mice in each group showed an increasing trend from days 1–5. The mean body weight of mice in both the L and L+S groups administered *L. brevis* 23017 by gavage was slightly higher than that of the control group. After administering *S. typhimurium* C7731 by gavage to mice in the S and L+S groups, the recorded body weight at days 6 and 8 was significantly less compared with that in the control group (*p* < 0.05). The decrease in body weight in the S group was more notable than that in the L+S group. The body weights of S and L+S groups were significantly lower than the weights of mice in the control and L groups (*p* < 0.05).

One and three days after infection, DAI scores showed an increasing tendency in the S group, with highly significant differences (*p* < 0.01) compared to the L+S group. However, the DAI scores of the L+S group displayed a decreasing tendency from 2 to 3 d after infection. The DAI scores of the control and the L groups were lower than those of the S group (Figure 1C). These results indicated that the DAI score of mice was significantly improved after administering *L. brevis* 23017 by gavage.

Compared with the S group, mice in the L+S group were more stable and able to drink and eat normally, but with reduced mobility. In contrast, mice in the S group exhibited a loss of appetite, were immobile, and abdominal swelling was present. Mice in the S group developed obvious diarrhea and malodorous stool; the symptoms in the L+S group were significantly better. The dissection results of mice on day 9 revealed swollen, congested, edematous, and easily ruptured intestinal morphology of mice in the S group. In contrast, the intestines of mice in the L+S group displayed only slight swelling and no areas of bleeding (Figure 1D).

The improvements in body weight, DAI score, and clinical symptoms demonstrated the protective effect of *L. brevis* 23017 on the intestinal damage in the mice.

### 3.2. L. brevis 23017 Decreases S. typhimurium C7731 in Tissues

The bacterial loads of *S. typhimurium* C7731 in the jejunum, liver, spleen, heart, and cecum were determined to verify the inhibitory effects of *L. brevis* 23017 against *S. typhimurium* C7731 challenge. In the jejunum, the viable counts of *S. typhimurium* C7731 in the L+S group was significantly reduced compared to the S group (*p* < 0.05). In the heart, liver, and spleen, the viable counts of *S. typhimurium* C7731 in the L+S group did not differ significantly compared to the S group. There was an increasing (but not statistically significant) tendency of the viable counts of *S. typhimurium* C7731 in the cecum of mice in the L+S group compared to the S group (Figure 1E). These results indicate that *L. brevis* 23017 inhibited the colonization of *S. typhimurium* C7731 in the tissues to some extent.

### 3.3. L. brevis 23017 Regulates Cytokines in Sera

**Figure 2:**
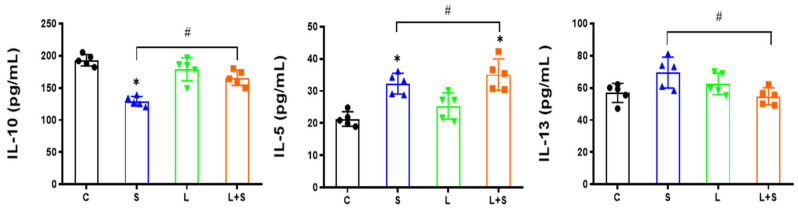
Measured serum inflammatory cytokines levels. * denotes a significant difference (*p* < 0.05) in the S and L+S control groups. # denotes a significant difference (*p* < 0.05) in the L group compared to the S group and L+S groups.

To better understand the protective efficacy of *L. brevis* 23017, cytokine levels in sera of mice were measured by ELISA. As shown in Figure 2, IL-5 concentrations significantly increased in the S and L+S groups compared to the control group (both *p* < 0.05). Significant increases were evident in IL-5 and IL-10 concentrations (*p* < 0.05) and IL-13 was significantly reduced (*p* < 0.05) in the L+S group compared with the S group. Oral *L. brevis* 23017 treatment increased serum concentrations of inflammatory cytokines in mice, resulting in milder inflammatory responses. These results suggest that *L. brevis* 23017 protected the intestine, regulated secretion of IL-10 and IL-13 in sera, and inhibited the intestinal inflammatory response.

### 3.4. L. brevis 23017 Prevents S. typhimurium C7731 Intestinal Infection by Improving Intestinal Immunity

Next, we determined the total SIgA content in small intestinal mucus to demonstrate the effect of *L. brevis* 23017 on the immune response. The content in the control, S, L, and L+S group was 43.72 ± 0.06, 58.82 ± 0.03, 43.81 ± 0.07, and 65.87 ± 0.18 ng/mL, respectively. The SIgA content in small intestinal mucus tended to increase in the S and L+S groups (*p* < 0.01) compared with the control group. However, oral feeding *L. brevis* 23017 did not increase the content of SIgA compared with the control group. SIgA content was slightly higher in the L group than in the control group, but was not significant. In addition, the SIgA secreted in the small intestinal mucus in the L+S group was significantly higher than in the S group (*p* < 0.05). These results revealed that *S. typhimurium* C7731 infection could increase the content of SIgA and that, after administering *L. brevis* 23017 by gavage, further improved the content of SIgA. The consequence was the promoted secretion of small intestinal mucus and facilitated secretion and action of intestinal antibodies.

### 3.5. L. brevis 23017 Inhibits Colitis Induced by S. typhimurium C7731 Infection and Maintains the Integrity of Tissue Morphology

**Figure 3:**
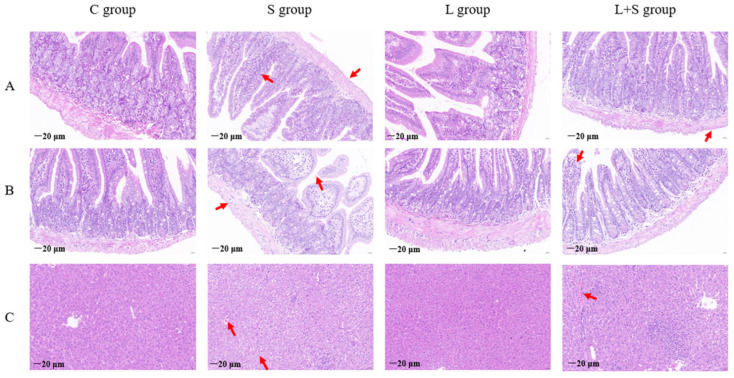
Histological images of the intestinal tract and organs (magnification 40×); The red arrow indicates the tissue lesion. (**A**) duodenum, (**B**) ileum, and (**C**) liver.

To determine if tissue integrity was affected by *S. typhimurium* C7731 infection, we monitored the morphology of samples. Hematoxylin and eosin staining of duodenum tissues revealed no differences in the control and L groups (Figure 3A). The most severe pathological changes were observed in the S group. Detachment of the mucosal layer of the villi, disruption of the mucosa, changes in cell morphology, and an increase in the intercellular space with a large accumulation of inflammatory cells and edema in the lamina propria cells of the villi were evident. In the L+S group, only a small portion of the villous tissue of the duodenum was shed compared to the S group. Histopathological results of the ileum are shown in Figure 3B. Similarly, no significant pathological changes were seen in the ileum of the control and L groups. In the S group, there was edema in the lamina propria cells of the ileal villi and a large number of infiltrating inflammatory cells. In addition, the mucosal layer of the villi was destroyed and the intestinal mucosal structure was partially lost. Slight inflammatory cell infiltration in the lamina propria cells of the intestinal villi was observed in the L+S group compared to the S group, but the morphological structure of the intestinal tissue was normal.

The liver is the most commonly involved organ affected after intestinal tract damage. As shown in Figure 3C, no significant pathological changes were observed in the liver tissue of the control and L groups. Hepatocytes in the S group were larger, with obvious nuclei, intercellular vacuoles, and inflammatory cell infiltration. Liver tissue of the L+S group showed slight cell swelling and partial inflammatory cell infiltration, indicating a protective effect of *L. brevis* 23017 on the integrity of liver tissue morphology.

### 3.6. L. brevis 23017 Effectively Increases the Antioxidant Activity of Intestinal Tissues Infected by S. typhimurium C7731

To detected oxidative stress levels in mice, we measured some indicators of the antioxidant enzyme system (MDA, SOD, GSH-Px, and T-AOC). The results are shown in Table 1. In the duodenum and jejunum, MDA activity was highest in the S group compared with the L+S group, followed by the L+S group, indicating that oxidative damage was most severe in the S group and less severe in the L+S group. Similarly, the control group had the highest SOD activity and the L+S group had higher SOD activity than the S group in the duodenum and jejunum (*p* < 0.05). GSH-Px activity was highest in the duodenum group, with no significant differences in the S and L+S groups. In the jejunum, GSH-Px activity was highest in the control group. T-AOC activity in the control group remained highest in the duodenum and jejunum, and was significantly different in the L group compared to the L+S group (*p* < 0.05). These results demonstrate the dysregulated antioxidant enzyme system in the mouse intestine, confirmed the occurrence of oxidative stress in the organism. The results further suggest that intestinal oxidative damage is an important pathogenic mechanism in intestinal disease caused by *S. typhimurium* C7731 infection.

### 3.7. L. brevis 23017 Regulates mRNA Expression of Genes Related to Intestinal Integrity and Inflammatory Mediators

**Figure 4:**
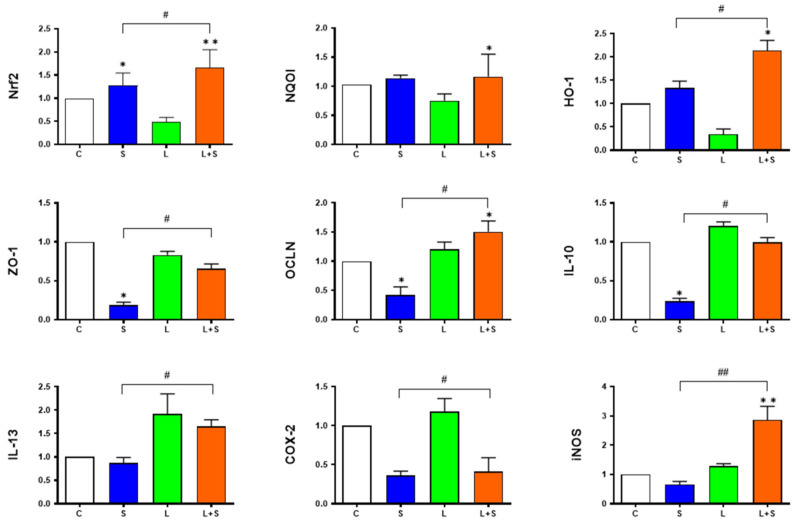
Expression of Nrf2 signaling pathway-related factors, intestinal immune-related factors, and intestinal integrity-associated genes in the duodenum. Means were considered to be significantly different at * *p* < 0.05 and ^#^ *p* < 0.05, and highly significant difference at ** *p* < 0.01 and ^##^ *p* < 0.01.

**Figure 5:**
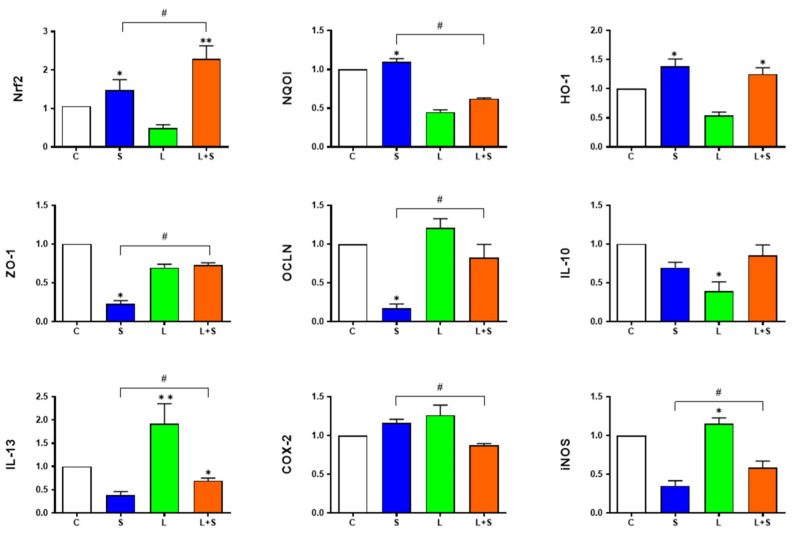
Expression of Nrf2 signaling pathway-related factors, intestinal immune-related factors, and intestinal integrity-associated genes in the jejunum. Means were considered to be significantly different at * *p* < 0.05 and ^#^ *p* < 0.05, and highly significantly different at ** *p* < 0.01.

To determine the maintenance of normal intestinal immune function through epithelial cell tight junction proteins and inflammatory mediators by *L. brevis* 23017, we measured the mRNA expression of ZO-1, OCLN, IL-10, IL-13, COX-2, and iNOS genes in the intestine. In the duodenum, compared with the control group, the expression of ZO-1, OCLN, and IL-10 genes were downregulated in the S group, with increased expression of the OCLN gene in the L+S group (*p* < 0.05). The expression of ZO-1 (*p* < 0.05), OCLN (*p* < 0.05), IL-10 (*p* < 0.05), COX-2 (*p* < 0.05), and iNOS (*p* < 0.01) genes were increased in the L+S group compared with the S group (Figure 4). In the jejunum, compared with the control group, the expression of ZO-1 and OCLN genes were downregulated in the S group (both *p* < 0.05). Compared with the S group, the expression of ZO-1, OCLN, and iNOS gens were upregulated in the L+S group (all *p* < 0.05), while the expression of COX-2 was lower in the L+S group than that in the S group (Figure 5).

### 3.8. L. brevis 23017 Increases mRNA Expression of Nrf2 Signaling Pathway-Related Factors

To assess the involvement of Nrf2-related antioxidant genes in vivo in the presence of *L. brevis* 23017, we measured the mRNA expression of Nrf2, NQO1, and HO-1 genes in the intestine. In the duodenum, the expression of Nrf2 (*p* < 0.01), NQO1 (*p* < 0.05), and HO-1 (*p* < 0.05) displayed a trend of significant increase in the L+S group compared to the control group. The expression of Nrf2 and HO-1 were elevated in the L+S group compared to the S group (both *p* < 0.05; Figure 4). In the jejunum, compared with the control group, the expression of Nrf2 (*p* < 0.01) and HO-1 (*p* < 0.05) in the L+S group showed an increasing trend. Compared with the S group, the expression of Nrf2 and NQO1 were significantly upregulated in the L+S group (both *p* < 0.05; Figure 5). These findings suggest that the positive regulation of *L. brevis* 23017 regulated the expression of the Nrf2 signaling pathway-related factor mRNAs.

### 3.9. L. brevis 23017 Upregulates mRNA Expression of Intestinal Immune-Related Factors

**Figure 6:**
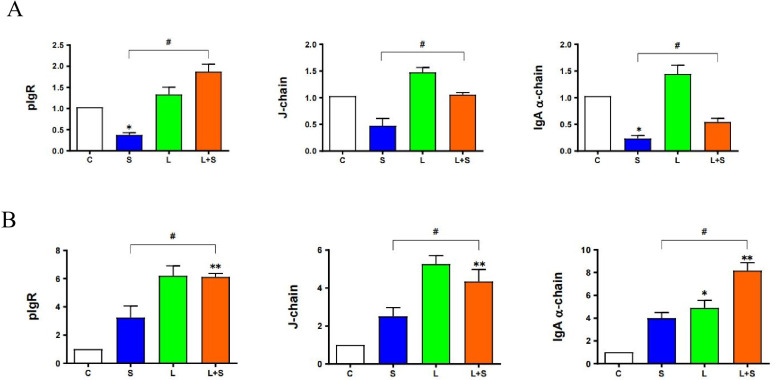
Expression of SIgA structure-related factors in the (**A**) duodenum and (**B**) jejunum. Means were considered to be significantly different at * *p* < 0.05 and ^#^ *p* < 0.05, and highly significantly different at ** *p* < 0.01.

The pIgR, J-chain, and IgA α-chain are structural factors in the composition of SIgA structure. Accordingly, we measured the expression of these three intestinal immune-related factors. In the duodenum, compared with the control group, the expression of pIgR in the L and L+S group showed an increasing trend. The expression of pIgR, J-chain, and IgA α-chain were significantly higher in the L+S group compared with the S group (all *p* < 0.05; Figure 6A). In the jejunum, compared with the control group, the expression of pIgR, J-chain, and IgA α-chain in the L+S group showed a highly significant upward trend (all *p* < 0.01). Compared with the S group, the expression of pIgR (*p* < 0.05), J-chain (*p* < 0.05), and IgA α-chain were both upregulated in the L+S group (Figure 6B). IL-13 is an important regulator of SIgA. Compared with the S group, the level of IL-13 in the L+S group was significantly increased in the duodenum and jejunum (*p* < 0.05; Figure 4 and Figure 5). These results further prove that *L. brevis* 23017 promoted the expression of SIgA.

### 3.10. L. brevis 23017 Protects Mice against Oxidative Stress Induced by S. typhimurium C7731 by Regulating the Nrf2 Signaling Pathway

**Figure 7:**
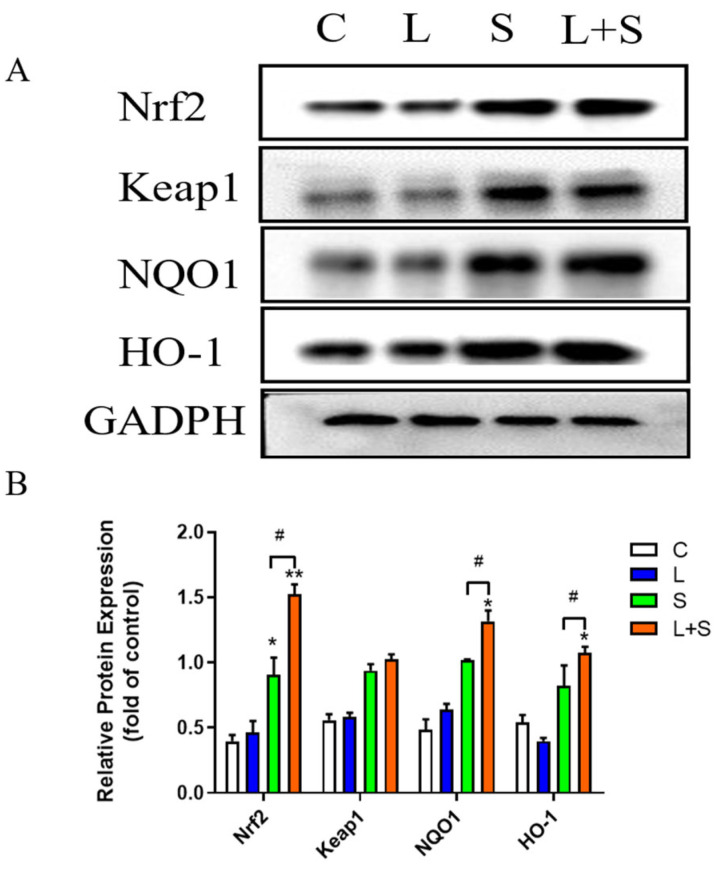
Detection of Nrf2-related signaling pathway proteins in jejunum. (**A**) Western blot detection results of Nrf2 signaling pathway proteins in the jejunum. (**B**) Expression of Nrf2 signaling pathway proteins relative to GADPH protein. Means were considered to be significantly different at * *p* < 0.05 and ^#^ *p* < 0.05, and highly significant difference at ** *p* < 0.01.

To evaluate the activation of the Nrf2 signaling pathway-related antioxidant proteins by *L. brevis* 23017 in the intestine, we measured the protein contents of Nrf2, Keap1, NQO1, and HO-1 in the intestines of mice. Signaling pathway proteins in the jejunum of *S. typhimurium* C7731-infected mice were detected by Western blot (Figure 7A). The results of the expression of the Nrf2 signaling pathway proteins relative to GADPH protein in the jejunum of the *S. typhimurium* C7731 infection model are shown in Figure 7B. In the *S. typhimurium* C7731 mouse intestinal infection model, upregulated expressions of Nrf2 (*p* < 0.05), HO-1, and NQO1 were evident in the S group compared with the control group. Compared with the control group, Nrf2 (*p* < 0.01), NQO1 (*p* < 0.05), and HO-1 (*p* < 0.05) protein expression levels were increased in the jejunum in the L+S group. NQO1, Nrf2, and HO-1 protein levels were upregulated in the jejunum in the L+S group compared to the S group (all *p* < 0.05), although there was no significant difference in the protein level changes of Keap1. These results indicate that *L. brevis* 23017 positively regulated the expression of antioxidant proteins to attenuate *S. typhimurium* C7731-induced oxidative stress in the intestine, confirming that the Nrf2 signaling pathway plays a critical role in the antibacterial effect of *L. brevis* 23017.

## 4. Discussion

*S. enterica* is an important foodborne pathogen globally. *S. typhimurium*, as one of the most common serotypes, mainly causes gastroenteritis to systemic infection in humans and animals [34,35]. However, there is obvious strain specificity. Although *L. brevis* has shown activity against *S. typhimurium* using in vitro assays, in vivo data are scant, and the relevant mechanism needs to be clarified. In this study, we selected *L. brevis* 23017, which can regulate the intestinal immunity of host animals and resist pathogenic infections [19]. We explored the protective effect of *L. brevis* 23017 against *S. typhimurium* C7731 infection in a mouse model. Our data indicate that *L. brevis* 23017 enhanced host defenses and regulated the Nrf2 signaling pathway to markedly reduce oxidative stress in mice caused by infection of *S. typhimurium* C7731.

*L. brevis* 23017 could inhibit *S. typhimurium* C7731 colonization in the intestine and restrict its translocation in organs. We observed a remarkable decrease in the number of *S. typhimurium* C7731 in the jejunum after administering *L. brevis* 23017 by gavage and a not particularly significant reduction in the viable number of *S. typhimurium* C7731 in the heart, liver, and spleen, consistent with the results of *L. plantarum* Tennozu-SU2 in a BALB/c mouse model featuring *S. typhimurium* infection [36]. In addition, other authors [37] also demonstrated that the probiotic *L*. *casei* (CRL-431) reduced the number of Salmonella infections of the intestine. However, compared with the other LAB strains, *L. brevis* 23017 was not strongly resistant to *S. typhimurium* C7731 colonization. Further studies are needed to analyze the antibacterial effect of *L. brevis* 23017.

We confirmed that *L. brevis* 23017 modulates intestinal immunity associated with facilitated intestinal mucus secretion of SIgA. SIgA is the first line of defense against infection. It promotes colonization of the mucosal surface by commensal microbiota and regulates immune homeostasis [38]. SIgA also reduces the virulence of microorganisms by altering the expression of the colonization factor and impairing motility, which neutralizes the bacterial toxin [39]. In the present study, compared with the *S. typhimurium* C7731-infected group, SIgA antibody levels were significantly increased after administering *L. brevis* 23017 by gavage. This finding is consistent with our previous study of *L. brevis* 23017 in response to *Yersinia enterocolitica* infection [20]. We further analyzed the transcription levels of pIgR, J-chain, and IgA α-chain, and observed significant increases. Cytokine analysis also revealed consistent elevations of the IL-5 and IL-13 cytokines, which are vital in intestinal immunity in regulating the secretion of SIgA [40,41]. *L. brevis* 23017 improved SIgA levels by regulating the level of IL-5, IL-13, pIgR, J-chain and IgA α-chain to enhance the immune function against *S. typhimurium* C7731 infection.

*L. brevis* 23017 attenuated intestinal oxidative damage by positively regulating antioxidant enzyme systems and the intestinal mucosal barrier. Gavage with *L. brevis* 23017 reversed the changes of the antioxidant enzymes SOD and GSH-Px, and T-AOC, and the oxidative stress marker MDA, with increased IL-10 levels. These findings are consistent with the report that *Bruguiera gymnorrhiza* (L.) Lam Fruit protects against dextran sulfate sodium-induced ulcerative colitis via the Keap1/Nrf2 pathway [42]. In the present study, the levels of the tight junction ZO-1 and OCLN, and anti-inflammatory cytokines were increased in the duodenum. The level of the inflammatory mediator COX-2 was decreased in the jejunum. Pathogenic bacteria affect intestinal epithelial cell barrier function by regulating the expression of tight junctions. Most probiotics can inhibit the increase in cell permeability by promoting the secretion of the OCLN, Claudin-1, and ZO-1 tight junction proteins as well as junctional adhesion molecules [14]. Normally, inflammation and mucosal barrier damage occur together, and cytokine secretion alters the regulation and expression of tight junction proteins, thus affecting barrier properties [15]. LAB can positively regulate the secretion of anti-inflammatory cytokines, such as IL-4 and IL-10, and prevents apoptosis of intestinal epithelial cells [43]. Our results supported these prior findings, suggesting that the ameliorative effect of *L. brevis* 23017 on oxidative stress induced by *S. typhimurium* C7731 is closely related to the production of the antioxidant enzyme system, tight junction proteins, and inflammatory mediators.

Our results show that *L. brevis* 23017 protects the intestine from oxidative damage and that its antioxidant activity is closely related to the activation of the Nrf2 signaling pathway. This pathway is pivotal in protecting against oxidative stress [44]. The genes encoding NQO1 and HO-1 are transcriptionally regulated by Nrf2; their activation maintains the redox homeostasis of cells [25,45]. The antioxidation mechanism of probiotic LAB involves a complex signaling network in which the Nrf2 signal pathway has been proven to be the key protective mechanism of probiotic antioxidant stress [26,46]. Several studies have demonstrated that Nrf2 is involved in probiotic-activated antioxidant defense using human and animal cell models in vitro [47,48]. In this study, we used qPCR and Western blot to detect the expression level of related antioxidant proteins and genes in the Nrf2 signaling pathway. Western blot results showed that the expressions of the Nrf2, NQO1, and HO-1 antioxidant proteins in the jejunum were significantly increased, in agreement with a previous study [49]. qPCR revealed that *L. brevis* 23017 had different degrees of activation of Nrf2 downstream gene expression. The mRNA expression levels of Nrf2, NQO1, and HO-1 in the duodenum showed an upward trend, consistent with the results of Nrf2-related antioxidant proteins in the jejunum. These results indicate that *L. brevis* 23017 could enhance the response of the Nrf2 signaling pathway, stimulate the activation of downstream genes and proteins, and promote the expression of Nrf2, NQO1, and HO-1 with the antioxidant capacity to attenuate the oxidative stress induced by *S. typhimurium* C7731.

**Figure 8:**
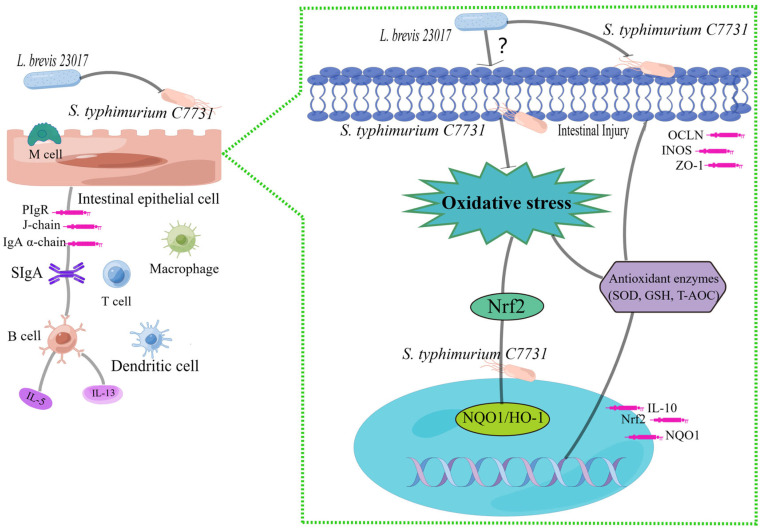
Potential mechanism of *L. brevis* 23017 probiotic effects against *S. typhimurium* C7731-induced intestinal injury. The figure was made using FIG DRAW (https://www.figdraw.com accessed on 20 March 2023).

We evaluated the mechanism of *L. brevis* 23017 on intestinal antioxidant damage from the perspective of the Nrf2 signaling pathway using a mouse *S. typhimurium* C7731 infection model. The potential mechanism of antioxidation of *L. brevis* 23017 is shown in Figure 8. However, whether the Nrf2 signaling pathway is linked to SIgA must still be demonstrated. Nrf2 acts as an important pathway for oxidative stress. The present findings demonstrate that *L. brevis* 23017 protection against intestinal damage is associated with the secretion of SIgA. Detection of inflammatory mediators revealed that the serum content of IL-13 decreased, while the serum levels of IL-13 in the duodenum and jejunum increased. The possible reason is that after administering *L. brevis* 23017 by gavage, the role of IL-13 in regulating inflammation is mainly reflected in the sera of mice, while the secretion of SIgA does not exert its effect in sera. The increase in IL-13 related to SIgA mainly plays a role in the intestinal tract. We further observed that the levels of COX-2, iNOS, and iNOS in the jejunum were elevated. COX-2 has anti-inflammatory and antioxidant activities, but their excessive release can lead to oxidative damage in the body. Therefore, we hypothesize that the jejunum is less sensitive to COX-2, while conversely the balance of COX-2 in the duodenum and jejunum ameliorates intestinal oxidative damage. In addition, the mRNA expression levels of NQO1 and HO-1 in jejunum showed a downward trend, but the mRNA expression level of the HO-1 gene had no statistical significance compared with the *S. typhimurium* C7731 infection group. The possible reason is that although the body can produce a small number of antioxidants through self-regulation in the late stage of infection, *S. typhimurium* C7731 can damage the intestinal tract more than the body’s repair capacity. Mu et al. [50] showed in their study on the control of lupus nephritis by alterations in the gut microbiota that the transcript levels of inflammatory mediators in the gut and serum were inconsistent after LAB treatment, which they attributed to gut-specific immunosuppression.

In the planned studies, Nrf2-knockout mice will be used to use an oxidative damage model to study the effect of Nrf2 gene deletion on *L. brevis* 23017 in the process of anti-intestinal oxidative damage. In addition, comprehensive analyses of the role and mechanism of LAB on intestinal antioxidant damage will be undertaken from the perspective of inhibiting the Nrf2 signaling pathway. We will also focus on identifying the fractions or products of LAB that are key to antioxidant activity. The findings could provide new therapeutic tools for the healthcare industry and for curing diseases associated with oxidative damage in the intestinal tract. Refinement of the above studies may establish a more precise link between intestinal oxidative stress and elevated intestinal mucosal immune antibody SIgA, which will also help develop new immune enhancers or adjuvants.

## 5. Conclusions

*L. brevis* 23017 slowed down the weight loss induced by *S. typhimurium* C3371 infection in mice and inhibited *S. typhimurium* C3371 colonization in the intestine and other organs. Notably, regulation of intestinal immunity by *L. brevis* 23017 was related to the promotion of SIgA secretion from intestinal mucus. In addition, *L. brevis* 23017 reduced oxidative damage by positively regulating lymphokines, antioxidant enzyme systems, and intestinal mucosal barrier genes, and by upregulating the expression levels of Nrf2, Keap1, NQO1 and HO-1 antioxidant proteins. These results suggest that *L. brevis* 23017 attenuates *S. typhimurium* C3371 induced intestinal oxidative stress through activation of the Nrf2 signaling pathway.

## Figures and Tables

**Table 1:** Antioxidant indicators in the duodenum and jejunum.

Intestinal Segments	Groups	MDA (nmol/mg Protein)	SOD (U/mg Protein)	GSH-Px (μmol/g Protein)	T-AOC (μmol/g Protein)
duodenum	C	4.25 ± 1.58	11.07 ± 4.67	10.16 ± 1.09	0.69 ± 0.08
	S	5.95 ± 1.09 ^#^	4.15 ± 1.93 *^#^	14.78 ± 3.84 *	0.25 ± 0.11 *^#^
	L	4.31 ± 1.82	10.97 ± 3.32	12.17 ± 1.49	0.43 ± 0.03
	L+S	4.54 ± 0.56 ^	10.52 ± 3.25 ^#^^^	14.85 ± 4.12 *^	0.39 ± 0.09 *^
jejunum	C	4.25 ± 1.38	10.77 ± 1.25	12.856 ± 0.86	0.62 ± 0.06
	S	5.88 ± 0.31 *^#^	4.51 ± 1.57 *^#^	7.81 ± 0.32 **^#^	0.36 ± 0.11 *^#^
	L	4.16 ± 0.97	9.27 ± 2.02 *	10.92 ± 0.57 *	0.42 ± 0.02
	L+S	4.31 ± 0.32 ^	8.75 ± 0.75 *^#^^	8.31 ± 3.21 *^#^^	0.51 ± 0.10 ^#^^

The levels of MDA, SOD, GSH-Px and T-AOC were used to evaluate antioxidant with duodenum and jejunum tissues. * *p* < 0.05, ** *p* < 0.01 for the control group comparing the S and L+S groups; # *p* < 0.05, L group compared with S and L+S groups; ^ *p* < 0.05, L+S compared with L groups; ^^ *p* < 0.01, L+S compared with L groups.

## Data Availability

The raw data supporting the conclusions of this article will be made available by the authors on request.

## Supplementary Materials

The following supporting information can be downloaded at: https://www.mdpi.com/article/10.3390/microorganisms12061135/s1, Table S1: The nutrient and energy composition of the standard mouse chow; Table S2: RT-qPCR primer sequences.
