# Supplementary material for: Molecular Mechanisms of Intestinal Protection by Levilactobacillus brevis 23017 against Salmonella typhimurium C7731-Induced Damage: Role of Nrf2
Source: Microorganisms. 2024 Jun 1;12(6):1135. doi: 10.3390/microorganisms12061135 (PMC11205325; doi:10.3390/microorganisms12061135)
Supplement: Supplementary file 1 [file microorganisms-12-01135-s001.zip › microorganisms-2977787-supplementary.pdf]

microorganisms

Supplementary Materials

Table S1. The nutrient and energy composition of the standard mouse chow.

Iron (Fe)	≥ 100 mg	Vitamin A	≥ 7000 IU	Vitamin B1	≥ 8 mg	Lysine	≥ 8.2 g
Manganese (Mn)	≥ 75 mg	Vitamin D	≥ 800 IU	Vitamin B2	≥ 10 mg	Egg + Cystine	≥ 5.3 g
Copper (Cu)	≥ 10 mg	Vitamin E	≥ 60 IU	Vitamin B6	≥ 6 mg	Arginine	≥ 9.9 g
Zinc (Zn)	≥ 30 mg	Vitamin K	≥ 3 mg	Vitamin B12	≥ 0.02 mg	Histidine	≥ 4 g

Raw material composition: corn, soybean meal, flour, bran, fish flour, salt, calcium hydrogen phosphate, rock flour, multivitamins, multiple trace elements, amino acids, etc.

Table S2. RT-qPCR primer sequences.

Gene	Primer	Primer sequence（5'–3'）
β-actin	β-actinF	CTGGAACGGTGAAGGTGA
	β-actinR	TTTGGAAAGGCAGGGACT
Nrf2	Nrf2F	AATGCATCTTATGCCGGTG
	Nrf2R	TCTTTGAATCTGTCCGAGAAT
NQO1	NQO1F	TGCCTTCCTTGACTTGCT
	NQO1R	TCCCGGCTTTACATCCTA
HO-1	HO-1F	AGCTGTTTCTGAGCCTCCAA
	HO-1R	CAAGACGGAAACACGAGACA
pIgR	pIgRF	CAGACATTAGCATGGCAGACTTCAA
	pIgRR	TGCCGAGTAGGCCATGTCAG
J Chain	J ChainF	TTTGTTAAGGCTGTCCTTGT
	J ChainR	AAGTGGAGCTGGAAGATCAG
α-chain	α-chainF	TGAGCGCTGGAACAGTGGCG
	α-chainR	TCAGGGCCAGCTCCTCCGAC
ZO-1	ZO-1F	AGGACACCAAAGCATGTGAG
	ZO-1R	GGCATTCCTGCTGGTTACA
OCLN	OCLNF	GCTGTGATGTGTGTGAGCTG
	OCLNR	GACGGTCTACCTGGAGGAAC
IL-13	IL-13F	AGACCAGACTCCCCTGTGCA
	IL-13R	TGGGTCCTGTAGATGGCATTG
IL-10	IL-10F	CGCAGCTCTAGGAGCATGTG
	IL-10R	GCTCTTACTGACTGGCATGAG
COX-2	COX-2F	ACCACACTTCATGCATCAGC
	COX-2R	ACTTGGAGCACAGGGGTCT
iNOS	iNOSF	CAGCTGGGCTGTACAAACCTT
	iNOSR	CATTGGAAGTGAAGCGTTTCG
